# Suppressive regulatory T cells and latent transforming growth factor-β-expressing macrophages are altered in the peritoneal fluid of patients with endometriosis

**DOI:** 10.1186/s12958-018-0325-2

**Published:** 2018-02-01

**Authors:** Tetsuro Hanada, Shunichiro Tsuji, Misako Nakayama, Shiro Wakinoue, Kyoko Kasahara, Fuminori Kimura, Takahide Mori, Kazumasa Ogasawara, Takashi Murakami

**Affiliations:** 1 Department of Obstetrics and Gynecology, Shiga University of Medical Science, Seta Tsukionowa-cho, Otsu, Shiga 520-2192 Japan; 2 Department of Pathology, Division of Pathology and Disease Regulation, Shiga University of Medical Science, Seta Tsukionowa-cho, Otsu, Shiga 520-2192 Japan; 3 Academia for Repro-Regenerative Medicine, Nonprofit Organization, 394-1 Higashi-Hinodono-cho, Ichijo-Shinmachi-Higashiiru, Kamigyo-ku, Kyoto, 602-0917 Japan

**Keywords:** Regulatory T cell, TGF-β, Latency-associated peptide, Endometriosis

## Abstract

**Background:**

Endometriosis is a known cause of infertility. Differences in immune tolerance caused by regulatory T cells (Tregs) and transforming growth factor-β (TGF-β) are thought to be involved in the pathology of endometriosis. Evidence has indicated that Tregs can be separated into three functionally and phenotypically distinct subpopulations and that activated TGF-β is released from latency-associated peptide (LAP) on the surfaces of specific cells. The aim of this study was to examine differences in Treg subpopulations and LAP in the peripheral blood (PB) and peritoneal fluid (PF) of patients with and without endometriosis.

**Methods:**

PB and PF were collected from 28 women with laparoscopically and histopathologically diagnosed endometriosis and 20 disease-free women who were subjected to laparoscopic surgery. Three subpopulations of CD4^+^ T lymphocytes (CD45RA^+^FoxP3^low^ resting Tregs, CD45RA^−^FoxP3^high^ effector Tregs, and CD45RA^−^FoxP3^low^ non-Tregs) and CD11b^+^ mononuclear cells expressing LAP were analyzed by flow cytometry using specific monoclonal antibodies.

**Results:**

Proportions of suppressive Tregs (resting and effector Tregs) were significantly higher in the PF samples of patients with endometriosis than in those of control women (*P* = 0.02 and *P* < 0.01, respectively) but did not differ between the PB samples of patients and controls. The percentage of CD11b^+^LAP^+^ macrophages was significantly lower in PF samples of patients with endometriosis than in those of controls (*P* < 0.01) but was not altered in the PB samples.

**Conclusion:**

Proportions of suppressive Tregs and LAP^+^ macrophages are altered locally in the PF of endometriosis patients.

**Electronic supplementary material:**

The online version of this article (10.1186/s12958-018-0325-2) contains supplementary material, which is available to authorized users.

## Background

Endometriosis, defined by the presence of endometrial glands and stroma outside the uterus, is a common, chronic, estrogen-dependent disease contributing to dysmenorrhea and infertility. One leading theory hypothesizes that peritoneal lesions are derived from steroid hormone-sensitive endometrial cells and tissues that are implanted on the peritoneal surface during retrograde menstruation, eliciting an inflammatory response. Although most women exhibit retrograde menstruation, affected women may suffer from immune dysfunction that interferes with the clearing of such lesions [1].

FoxP3-expressing CD4^+^ regulatory T cells (Tregs) play an indispensable role in the maintenance of self-tolerance and immune homeostasis [2] and are involved in various human diseases, such as autoimmune diseases, allergies, and cancer [3–5]. FoxP3^+^ cells, however, are heterogeneous in function and phenotype. Miyara et al. suggested that human FoxP3-expressing CD4^+^ Tregs can be separated into three functionally and phenotypically distinct subpopulations: 1) CD45RA^+^FoxP3^low^ resting Tregs (rTregs), 2) CD45RA^−^FoxP3^high^ effector Tregs (eTregs), and 3) cytokine-secreting CD45RA^−^FoxP3^low^ non-Tregs (non-Tregs) [6, 7]. rTregs and eTregs have been shown to be suppressive Tregs, whereas non-Tregs are non-suppressive. Although rTregs do not exhibit proliferative potential in vitro, upon antigenic stimulation, they acquire an eTreg phenotype, i.e., FoxP3 is upregulated, followed by proliferation and eventual differentiation into eTregs. In humans, conflicting results have been obtained regarding whether populations of Tregs in the peritoneal fluid (PF) or peripheral blood (PB) differ significantly between patients with endometriosis and controls [8–10]. Moreover, the association between endometriosis and Treg subpopulations has not been well studied. Thus, in this study, we focused on assessing the association of each Treg subpopulation with endometriosis.

Transforming growth factor-β (TGF-β) induces FoxP3^+^ Tregs and inhibits the proliferation of immune cells and cytokine production via FoxP3-dependent and -independent mechanisms [11]. TGF-β is secreted as an inactive form that is trapped by latency-associated peptide (LAP) to form the small latent complex. The addition of the latent TGF-β-binding protein forms the large latent complex, which is deposited onto the extracellular matrix. After being released from LAP, activated TGF-β plays important roles in the control of cell proliferation, differentiation, and apoptosis [12]. According to several studies, a strong association between TGF-β levels in PF and endometriosis has been observed [13]. PF mainly comprises macrophages, with smaller numbers of lymphocytes, natural killer cells, and mesothelial cells [14, 15]. However, the contribution of activated TGF-β released from macrophages remains unclear, as the surface expression of LAP on the macrophages of endometriosis patients has not yet been determined.

Therefore, in the present study, we assessed the immunotolerance of patients with endometriosis by analyzing Treg subpopulations and LAP expression in macrophages and monocytes derived from the PF and PB, respectively, of patients with endometriosis and controls.

## Methods

### Patients

This single-center study was approved by the Ethics Committee of Shiga University of Medical Science (approval number: 26–217) prior to patient recruitment. Patients were recruited the day before surgery when they were hospitalized in the University Hospital. All participants were Japanese, and only women who still exhibited a menstrual cycle were recruited; patients who were menstruating at the time of admission were not recruited. All patients accepted the recruitment, and they were distributed into endometriosis group or control group based on the findings of laparoscopic surgery and post-surgical histopathology. Five patients were excluded (4 due to insufficient volume of the PF and 1 due to an acute inflammatory finding during surgery and a diagnosis of chlamydia peritonitis after surgery), and a total of 28 women diagnosed with endometriosis (mean age: 34.3 years, range: 23–46 years) were enrolled. All patients with endometriosis were classified as stage I–IV according to the revised American Society for Reproductive Medicine classification [16]. In the endometriosis group, 12 patients were in the proliferative phase, and 16 were in the secretory phase. Patients without visible endometriotic foci and pelvic inflammation who underwent laparoscopic excision of benign ovarian tumors and uterine myomas or diagnostic laparoscopy for infertility were assigned to the control group. A total of 20 women were enrolled in the control group (mean age: 36.1 years, range: 19–46 years). In the control group, 10 were in the proliferative phase, and 10 were in the secretory phase. Samples in the proliferative phase were collected on days 5–10 of the menstrual cycle, and samples in the secretory phase were collected on days 3–10 after ovulation. None of the patients were being treated with pharmacological medications, such as gonadotropin-releasing hormone agonists, low-dose estrogen and progestin, or dienogest, and all patients were free from other acute or chronic inflammatory diseases. Table 1 shows a summary of patient characteristics, including phase of the menstrual cycle and stage of endometriosis.

**Table 1:** Summary of patient characteristics

		Endometriosis		Control	*P value*
		(*n* = 28)		(*n* = 20)	
Age in years, mean (range)		34.3 (23–46)		36.1 (19–36)	0.13
BMI, mean (range)		20.3 (17.3–24.0)		21.0 (18.5–25.7)	0.45
Gravida, mean (range)		0.68 (0–2)		1.0 (0–4)	0.67
Para, mean (range)		0.39 (0–2)		0.75 (0–3)	0.46
Smokers		none		1	Not applicable
Stage of Menstrual Cycle	Proliferative	12		10	Not applicable
	Secretory	16		10	Not applicable
Stage of Endometriosis		I	7	Not applicable	
		II	6		
		III	11		
		IV	4		

Differences between groups were analyzed by Mann-Whitney *U*-tests

### Sample collection

PB samples were obtained from patients and controls and were added to heparin-containing tubes within 24 h of surgery. PB mononuclear cells (PBMCs) were isolated from residual blood cells by density gradient centrifugation using Ficoll-Paque PLUS (GE Healthcare, Little Chalfont, UK). Cells were washed twice with phosphate-buffered saline by centrifugation at 400×*g* for 10 min. PF was aspirated by a laparoscopic procedure and added to heparin-containing tubes. The supernatant from the ascites was collected by centrifugation at 400×*g* for 5 min. Mononuclear cells (MNCs) were isolated following the same method used for PBMCs. All samples were transferred to our laboratory within 30 min of collection, and the aforementioned procedure was started immediately.

### Monoclonal antibodies

Monoclonal antibodies against CD4-FITC (RPA-T4), CD11b-APC (ICRF44), CD45RA-APC (5H9), and FoxP3-PE (259D/C7) were purchased from Becton Dickinson (Franklin Lakes, NJ, USA). LAP-PE (clone 27,232) was purchased from R&D Systems (Minneapolis, MN, USA). Mouse IgG1-FITC, mouse IgG1-PE, and mouse IgG1-APC were purchased from eBioscience (San Diego, CA, USA) and were used as isotype-matched negative controls. Human Fc receptor blocker was purchased from Becton Dickinson. Ethidium monoazide bromide was purchased from Molecular Probes, Inc. (Eugene, OR, USA).

### Flow cytometry analysis

**Fig. 1:**
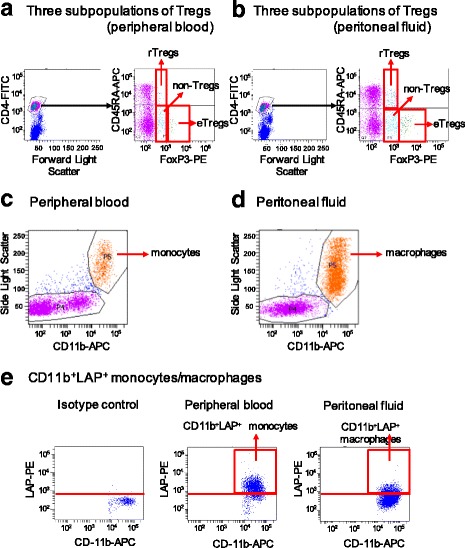
Determination of frequencies of Tregs and LAP^+^ macrophages and monocytes. **a**, **b** Gating of CD4^+^ T lymphocytes and determination of the proportions of three subpopulations in the peripheral blood (**a**) and peritoneal fluid (**b**): CD45RA^+^FoxP3^low^ resting Tregs (rTregs), CD45RA^−^FoxP3^high^ effector Tregs (eTregs), and CD45RA^−^FoxP3^low^ non-Tregs (non-Tregs). **c**, **d** Side light scattering and staining with CD11b gating to detect CD11b^+^ monocytes and macrophages from the peripheral blood (**c**) and peritoneal fluid (**d**). **e** Staining with isotype control, CD11b^+^LAP^+^ monocytes from the peripheral blood, and CD11b^+^LAP^+^ macrophages from the peritoneal fluid

For Treg assays, PBMCs and MNCs were blocked with human Fc receptor blocker for 10 min on ice and stained for 20 min on ice in the dark using antibodies against CD4 and CD45RA. After washing, the cells were stained with ethidium monoazide bromide for 20 min on ice and permeabilized using a FoxP3 Staining Kit (Becton Dickinson), according to the manufacturer’s protocol. Cells were analyzed using a FACS Aria flow cytometer (BD Biosciences, CA, USA), and 20,000 CD4^+^ cells were obtained (Fig. 1a and b). For monocyte and macrophage LAP staining assays, PBMCs and MNCs were stained to detect CD11b and LAP. Cells were analyzed using a FACS Aria flow cytometer, and 20,000 CD11b^+^ cells were obtained (Fig. 1c and d).

### Evaluation of TGF-β concentration in the PF

The frozen plasma samples of the PF were preserved at − 80 °C until analysis. Subsequently, the samples were sent to SRL Inc. (Tokyo, Japan) for evaluation of the concentration of TGF-β1 by enzyme-linked immunosorbent assay (ELISA) using a Quantikine human TGF-β1 immunoassay (R&D Systems; Minneapolis, MN, USA), which was performed according to the manufacture’s recommendation.

### Statistical analysis

Statistical analyses were performed using GraphPad Prism version 6 (GraphPad Software, Inc., La Jolla, CA, USA). Continuous variables were analyzed using Mann-Whitney *U*-tests. Fisher’s exact tests were performed for comparisons between two groups. Differences with *P* < 0.05 were considered statistically significant.

## Results

### Treg subpopulations in the PF and PB of patients with endometriosis and controls

**Fig. 2:**
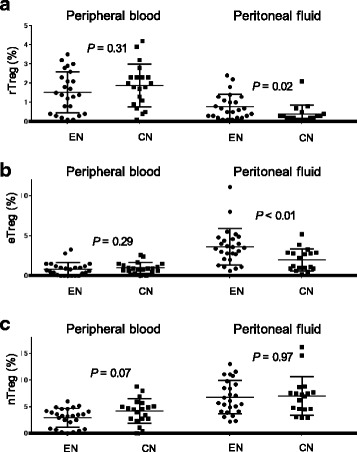
Proportion of Tregs in peripheral blood and peritoneal fluid of patients with endometriosis and controls. Proportions of CD45RA^+^FoxP3^low^ (rTregs) (**a**), CD45RA^−^FoxP3^high^ (eTregs) (**b**), and CD45RA^−^FoxP3^low^ (non-Tregs) cells (**c**) among the CD4^+^ population in the peripheral blood and peritoneal fluid of patients with endometriosis (EN, *n* = 28) and controls (CN, *n* = 10). Horizontal bars represent medians. Differences between groups were analyzed by Mann-Whitney *U*-tests

We gated CD4^+^ T lymphocytes and determined the proportions of three subpopulations of cells in the PB and PF: CD45RA^+^FoxP3^low^ rTregs, CD45RA^−^FoxP3^high^ eTregs, and CD45RA^−^FoxP3^low^ non-Tregs (Fig. 1a and b). We did not detect cyclical changes in these subpopulations in patients with endometriosis or controls (Table 2). FACS analysis revealed that the frequencies of rTregs and eTregs in the PF were significantly higher in the endometriosis group (medians: 0.60% and 3.4%, respectively) than in the control group [medians: 0.25% (*P* = 0.02) and 1.7% (*P* < 0.01), respectively]; however, these did not differ between the two groups in the PB [median rTregs: 1.4% (endometriosis) and 1.9% (control), *P* = 0.31; median eTregs: 0.70% (endometriosis) and 0.90% (control), *P* = 0.29] (Fig. 2a and b). There were no significant differences in non-Tregs between patients and controls in either the PB or PF (Fig. 2c). Significant differences were also observed in comparisons based on the menstrual cycle stage (Table 3). Comparison of rTregs and eTregs in the PF with severity of endometriosis (rASRM stage I and II vs. III and IV) showed no significant difference between the two groups [median rTregs: 0.75% (stage I and II) and 0.65% (stage III and IV), *P* = 0.59; median eTregs: 2.8% (stage I and II) and 3.6% (stage III and IV), *P* = 0.28].

**Table 2:** Comparison of Treg subpopulations, LAP^+^CD11b^+^ monocytes, and TGF-β between proliferative and secretory phases in each group (mean ± SD)

Peritoneal fluid											
	Phase	rTregs (%)	*P*	eTregs (%)	*P*	nonTregs (%)	*P*	LAP+CD11b + Macrophages	*P*	TGF-β	*P*
Endometriosis	Proliferative (*n* = 12)	0.73 ± 0.56	ns	3.9 ± 1.7	ns	7.7 ± 2.7	ns	13.0 ± 14.4	ns	1.12 ± 0.57	ns
	Secretory (*n* = 16)	0.82 ± 0.75		3.1 ± 2.8		6.0 ± 3.0		12.1 ± 16.4		1.09 ± 0.32	
Control	Proliferative (*n* = 10)	0.47 ± 0.60	ns	2.2 ± 1.5	ns	5.8 ± 2.1	ns	80.0 ± 19.7	ns	0.85 ± 0.26	ns
	Secretory (*n* = 10)	0.30 ± 0.24		1.8 ± 1.3		8.6 ± 1.6		81.3 ± 17.3		0.79 ± 0.26	
Peripheral blood											
	Phase	rTregs (%)	*P*	eTregs (%)	*P*	nonTregs (%)	*P*	LAP+CD11b + Monocytes	*P*		
Endometriosis	Proliferative (*n* = 12)	1.3 ± 0.61	ns	1.2 ± 2.2	ns	2.4 ± 1.9	ns	83.5 ± 16.7	ns		
	Secretory (*n* = 16)	2.1 ± 1.6		0.9 ± 1.0		3.0 ± 1.8		80.3 ± 19.7			
Control	Proliferative (*n* = 10)	1.3 ± 2.6	ns	1.2 ± 0.8	ns	4.4 ± 2.6	ns	79.2 ± 19.1	ns		
	Secretory (*n* = 10)	0.8 ± 2.8		0.8 ± 0.4		3.9 ± 2.2		85.9 ± 11.5			

Differences between groups were analyzed by Mann-Whitney *U*-tests. ns, not significant

**Table 3:** Comparison of Treg subpopulations, LAP^+^CD11b^+^ monocytes, and TGF-β concentrations in proliferative and secretory phases (mean ± SD) between endometriosis and control groups

Peritoneal fluid										
Proliferative	rTregs (%)	*P*	eTregs (%)	*P*	nonTregs (%)	*P*	LAP+CD11b + Macrophages	*P*	TGF-β	*P*
Endometriosis (*n* = 12)	0.73 ± 0.56	< 0.05	3.9 ± 1.7	< 0.05	7.7 ± 2.7	ns	13.0 ± 14.4	< 0.05	1.12 ± 0.57	< 0.05
Control (*n* = 10)	0.47 ± 0.60		2.2 ± 1.5		5.8 ± 2.1		80.0 ± 19.7		0.85 ± 0.26	
Secretory	rTregs (%)	*P*	eTregs (%)	*P*	nonTregs (%)	*P*	LAP+CD11b + Macrophages	*P*	TGF-β	*P*
Endometriosis (*n* = 16)	0.82 ± 0.75	< 0.05	3.1 ± 2.8	< 0.05	6.0 ± 3.0	ns	12.1 ± 16.4	< 0.05	1.09 ± 0.32	< 0.05
Control (*n* = 10)	0.30 ± 0.24		1.8 ± 1.3		8.6 ± 1.6		81.3 ± 17.3		0.79 ± 0.26	
Peripheral blood										
Proliferative	rTregs (%)	*P*	eTregs (%)	*P*	nonTregs (%)	*P*	LAP+CD11b + Monocytes	*P*		
Endometriosis (*n* = 12)	1.3 ± 0.61	ns	1.2 ± 2.2	ns	2.4 ± 1.9	ns	83.5 ± 16.7	ns		
Control (*n* = 10)	1.3 ± 2.6		1.2 ± 0.8		4.4 ± 2.6		79.2 ± 19.1			
Secretory	rTregs (%)	*P*	eTregs (%)	*P*	nonTregs (%)	*P*	LAP+CD11b + Monocytes	*P*		
Endometriosis (*n* = 12)	2.1 ± 1.6	ns	0.9 ± 1.0	ns	3.0 ± 1.8	ns	80.3 ± 19.7	ns		
Control (*n* = 10)	0.8 ± 2.8		0.8 ± 0.4		3.9 ± 2.2		85.9 ± 11.5			

Differences between groups were analyzed by Mann-Whitney *U*-tests. ns, not significant

### CD11b^+^LAP^+^ MNCs in the PF and PB of patients with endometriosis

**Fig. 3:**
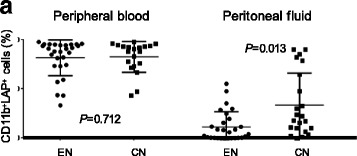
CD11b^+^LAP^+^ cells in peripheral blood and peritoneal fluid of patients with endometriosis and controls. Endometriosis, EN (*n* = 28); control, CN (*n* = 20). Horizontal bars represent medians. Columns and vertical bars indicate the 25th–75th percentiles and 10th–90th percentiles, respectively. Differences between groups were analyzed by Mann-Whitney *U*-tests

PBMCs and MNCs were examined using side light scattering and analysis of CD11b expression to detect monocytes and macrophages by flow cytometry (Fig. 1c and d). We then determined the frequencies of LAP^+^ cells among CD11b^+^ monocytes and macrophages in the PB and PF (Fig. 1e). We did not detect cyclical changes in the proportions of LAP^+^ macrophages in patients with endometriosis or controls (Table 2). We found that the proportion of LAP^+^ macrophages in the PF was significantly lower in patients with endometriosis (median: 6.2%) than in controls (median: 21.2%, *P* < 0.01), as shown in Fig. 3. There were no significant differences in the proportion of LAP^+^ monocytes in the PB of patients with endometriosis and controls (median: 89.4% and 89.2%, respectively; *P* = 0.86). Significant differences were also observed in comparisons based on the menstrual cycle stage (Table 3). Comparison of LAP^+^ macrophages in the PF with severity of endometriosis (rASRM stage I and II vs. III and IV) showed no significant difference between the two groups [median 31.1% (stage I and II) and 13.7% (stage III and IV), *P* = 0.23].

### Evaluation of TGF-β concentration in the PF

The concentration of TGF-β in the PF was significantly increased in patients with endometriosis (median 1.08 ng/mL) compared with that in the controls (median 0.75 ng/mL, *P* < 0.01, as shown in Additional file 1: Figure S1).

## Discussion

In the present study, we demonstrated that frequencies of rTregs and eTregs in the PF were higher in patients with endometriosis than in controls, but this pattern was not observed in the PB. In *Papio anubis*, induction of endometriosis alters the peripheral and endometrial populations of Tregs [17], whereas in humans, several studies have shown that Tregs are present in eutopic and ectopic endometrial tissues [18, 19] and that *FOXP3* mRNA levels are elevated in human endometriotic lesions [20]. These findings and our results together suggest that in humans, elevations in rTregs and eTregs in patients with endometriosis are not systemic but are locally induced in the peritoneal cavity. In contrast, in a recent report, there were no significant differences in Treg subpopulations in the PF of individuals with or without endometriosis [21]. The discrepancy in this result from ours may be caused by the different methods used to analyze Treg subpopulations: in this previous study, specimens were stored at − 80 °C before analysis by flow cytometry, and all specimens were obtained from patients with severe endometriosis. It has been reported that cryopreservation and thawing of specimens alter the phenotype and function of Tregs [22, 23].

Our results also demonstrated low LAP expression on the surfaces of macrophages in the PF of patients with endometriosis. Macrophage numbers are increased in the peritoneal cavities of women with endometriosis [24, 25]; therefore, the present study focused on macrophages in the PF. Disruption of the noncovalent interactions between LAP and TGF-β by either conformational changes or the complete dissociation of LAP can be induced under extreme conditions, e.g. extreme pH, heat, or the presence of serine protease plasmin, thrombospondin-1, reactive oxygen species, or integrin αγβ6 [26–28]. Active TGF-β can be released following this disruption, limiting tissue repair and promoting the development of tissue fibrosis [29–31]. Elevated concentrations of TGF-β in the PF of patients with endometriosis have been repeatedly reported [13, 32], and this result was further confirmed in our study (Additional file 1: Figure S1). Our results suggest that activated TGF-β released from the latent complex on macrophage surfaces may act to decrease LAP expression and increase the concentration of TGF-β in the PF of patients with endometriosis.

The overexpression of TGF-β has been implicated in fibrotic diseases of the kidney, liver, lung, skin, and other organs, and trapping TGF-β by increasing the expression of LAP in organ-specific cells leads to the suppression of fibrosis in the skin, hepatic tissue, and renal tissue [29, 33, 34]. Thus, higher levels of TGF-β may account for the peritoneal tissue fibrosis found in patients with endometriosis and are related to the severity of endometriosis [35]. In 2015, the results of a phase II clinical trial of fresolimumab, a neutralizing antibody against TGF-β1, β2, and β3, for skin sclerosis were reported. Fresolimumab treatment did not change the thickness of the dermis, but decreased the number of myofibroblasts [36]. Clinical studies using molecular targeted drugs for treatment of endometriosis have not yet been conducted, but monoclonal antibodies against TGF-β and LAP may be a promising approach for control of the disease.

As this is a cross-sectional study, it is difficult to determine whether the changes in TGF-β, LAP^+^ macrophages and suppressive Tregs are the cause or result of endometriosis; moreover, the trigger for LAP release also remains unknown. Though our data suggest a potential source of activated TGF-β, namely the reduced LAP expression on the surfaces of macrophages, TGF-β is produced by several types of cells including immune cells [37], and it has been reported that the peritoneum is a source of TGF-β in patients with endometriosis [38]. Additionally, as rASRM classification is based on macroscopic findings, potential endometriosis patients might be included in the control group, resulting in some data overlap between the groups. Regarding the limitation of the laboratory method, it is difficult to completely eliminate leucocytes from the PF derived from the PB due to contamination during operation. Furthermore, the participants of this study may include patients with dysmenorrhea symptoms such as pelvic pain and excessive menstruation, and infertility. There are some reports that endometriosis is related to infertility, pain, socioeconomic condition, stress, nickel allergy, vitamin D intake [39–42], and these potential confounding factors cannot be excluded completely with only the variabilities that we adjusted. However, owing to the small sample size of this study, it was difficult to adjust for all of these factors. We believe that such studies could clarify the pathophysiology of endometriosis and establish new treatment modalities.

## Conclusions

Our results revealed increases in the proportions of rTregs and eTregs in the PF of women with endometriosis, as well as reduced expression of LAP on macrophages, resulting in the release of activated TGF-β. These observations may be associated with suppressed immune responses, which may initiate and promote endometriosis in the pelvic cavity. Further studies on the relevance of Tregs and LAP-TGF-β are needed, and elucidation of this cascade may lead to the establishment of effective approaches for the control of endometriosis.

## Additional file

Additional file 1: Figure S1. Transforming growth factor-β (TGF-β) concentrations in peritoneal fluid of patients with endometriosis and controls. Concentrations determined by enzyme-linked immunosorbent assay. Endometriosis, EN (*n* = 28); control, CN (*n* = 20). Horizontal bars represent medians. Columns and vertical bars indicate the 25th–75th percentiles and 10th–90th percentiles, respectively. Differences between groups were analyzed by Mann-Whitney *U*-tests. (PDF 14 kb)

## Abbreviations

eTregs: CD45RA^−^FoxP3^high^ effector Tregs
LAP: Latency-associated peptide
MNCs: Mononuclear cells
non-Tregs: CD45RA^−^FoxP3^low^ non-Tregs
PB: Peripheral blood
PBMCs: Peripheral blood mononuclear cells
PF: Peritoneal fluid
rTregs: CD45RA^+^FoxP3^low^ resting Tregs
TGF-β: Transforming growth factor-β
Tregs: Regulatory T cells
