# Supplementary figures and images for: Suppressive regulatory T cells and latent transforming growth factor-β-expressing macrophages are altered in the peritoneal fluid of patients with endometriosis
Source: Reprod Biol Endocrinol. 2018 Feb 1;16:9. doi: 10.1186/s12958-018-0325-2 (PMC5796574; doi:10.1186/s12958-018-0325-2)

# Peritoneal fluid

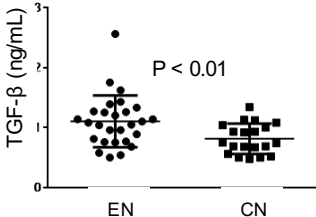

Supplement: Additional file 1: Figure S1. — Transforming growth factor-β (TGF-β) concentrations in peritoneal fluid of patients with endometriosis and controls. Concentrations determined by enzyme-linked immunosorbent assay. Endometriosis, EN (n = 28); control, CN (n = 20). Horizontal bars represent medians. Columns and vertical bars indicate the 25th–75th percentiles and 10th–90th percentiles, respectively. Differences between groups were analyzed by Mann-Whitney U-tests. (PDF 14 kb) [file 12958_2018_325_MOESM1_ESM.pdf]
